# Rapid Apical Healing with Simple Obturation Technique in Response to a Calcium Silicate-Based Filling Material

**DOI:** 10.1155/2022/6958135

**Published:** 2022-05-09

**Authors:** Bahaa AlBakhakh, Aqeel Al-Saedi, Riad Al-Taee, Mohammed Nahidh

**Affiliations:** ^1^Department of Conservative Dentistry, College of Dentistry, University of Almaaqal, Basrah, Iraq; ^2^Department of Oral Medicine, College of Dentistry, University of Basrah, Basrah, Iraq; ^3^Department of Maxillofacial Surgery, College of Dentistry, University of Almaaqal, Basrah, Iraq; ^4^Department of Orthodontics, College of Dentistry, University of Baghdad, Baghdad, Iraq

## Abstract

**Background:**

Root canal sealers with high alkaline (pH more than 11) and bioactive properties (release ions) are more effective for healing the apical area and preventing reinfection in root canals. Pure calcium silicate-based bioceramic premixed injectable sealers lead to change the obturation technique because it depends on maximizing amount of sealer and single cone gutta-percha as a carrier. This retrospective clinical study aimed at demonstrating the effect of single cone gutta-percha with a calcium silicate-based filling, used to fill the root canal in a single visit, on the apical healing with different lesion size.

**Materials and Methods:**

195 patients needing root canal treatment were treated at the Al-Jazaer private clinic in southern Iraq. Cases were obtained during the 2017–2021 period (retrospective study). All patients were treated with a simple hydraulic condensation technique that used calcium silicate-based filling materials as the sealer with single cone gutta-percha as the carrier, with a minimum follow-up period of approximately one year. Both treatment- and patient-related factors were evaluated. The clinical outcomes depended on classification with respect to complete (success) or incomplete (failure) healing. Statistical analysis was performed using a chi-square test to compare different related factors.

**Results:**

Radiographs for 195 patients were examined, and the average follow-up time was 20.44 months. The complete healing success rate was 164 (84.1%), and 31(15.9%) patients were not healed. The highly significant success rate was 88.7% for the initial treatment compared with 63.9% for retreatment. Small and medium lesions (<5 mm diameter) had a significant success rate compared to large lesions (>5 mm diameter). Sealer extrusion was found in 68.7% of cases, but it did not have any significant effect on the treatment result.

**Conclusions:**

The bioceramic calcium silicate-based filling sealer used with single-cone gutta-percha led to increased success of apical healing with a simple hydraulic condensation technique without multiple visit treatments and premedication material.

## 1. Introduction

Root canal sealers that are high in alkaline (pH more than 11) and bioactive properties (release ions) are able to heal the apical area more effectively and prevent reinfection in root canals [1, 2].

The bioceramic sealer—TotalFill—from FKG, premixed with an injectable sealer with a chemical composition of calcium phosphate, tricalcium silicate, colloidal silica, dicalcium silicate, and calcium hydroxide [3] is highly alkaline (pH more than 12), strongly antibacterial, with no polymerization shrinkage, is a bioactive material, insoluble against tissue body fluid after setting, with tight apical sealing, biocompatible, reacts with water (tissue body fluid in dentinal tubules), and completes the setting to form apatite-layer-like bone tissue [4–6].

The introduction of new bioceramic sealers has changed the obturation technique. Bhandi et al. concluded that neither techniques of obturation—cold lateral condensation or warm gutta-percha—provide a void-free and adequate seal root canal obturation due to shrinkage of gutta-percha when examined using micro-CT because these techniques depended on gutta-percha as a main filling obturation material [7] while new hydraulic condensation technique depend on sealer as a main obturation material with a gentle pumping action, are pushed into the complex canal system, which if properly cleaned, allows low-viscosity cements to reach even very small spaces with good sealing apically [6].

Researchers have evaluated the antibacterial efficacy of this sealer in comparison with other types of sealer, concluding that bioceramic calcium silicate-based materials are superior in terms of antibacterial properties [8–10] and that root canal treatments are successful, with the teeth having adequate clinical function [11].

Sodium hypochlorite (NaOCl) is the most effective irrigants used for root canal treatments, and at a concentration of 5.25%. It appears to dissolve necrotic tissue and kill most types of biofilm [12–14].

Ethylene diamine tetra acetic acid (EDTA) is used after NaOCl as the final irrigant [15–18] and is acidic in nature and slightly alkaline in neutral. Its main advantage is that it can remove smear layers in dentinal tubules [19, 20]. These layers contain various persistent anaerobic bacteria that require removal.

There are different methods for diagnosing periapical lesions to differentiate between granuloma and cystic lesions in apical area. The standard one is the histopathological diagnosis yet, unfortunately, it is difficult to take biopsy from each patient. The most accurate diagnosis means are MRI and CBCT images which are difficult to be available in each dental clinic that need special equipment. This equipment is costly and has longer scanning time. Recently, radiation-free, noninvasive ultrasonic imaging can give accurate diagnosis [21]. Unfortunately, the technique is not available in our clinic. Also, this technique cannot distinguish between various types of periapical cysts and difficult to give accurate diagnosis when the cortical plate is eroded; therefore, digital periapical radiograph is still common with low dose of radiation and easy manipulation [21].

The aim of the present study is to evaluate the periapical healing in response to a calcium silicate-based sealer using a hydraulic condensation obturation technique.

## 2. Materials and Methods

One hundred and ninety-five patients were treated at Al-Jazaer private dental clinic in Basrah city from March 2017 until March 2020 by two endodontists in a single visit.

### 2.1. Selection Criteria

Patients were selected on the basis of the following criteria:

1. Cases without apical surgery including initial or retreatment patients.
2. Digital radiographs and records documenting both diagnosis.

### 2.2. Exclusion Criteria

Cases were excluded from the research if there was evidence of the following clinical signs and symptoms: crack propagation, perforation, excessive periodontal disease, and vertical root fracture.

## 3. Methods

The procedure began with local anesthesia administration; then, the tooth was isolated with a rubber dam. An access opening preparation was made for retreatment, and the old gutta-percha was removed mechanically with retreatment D-Race files (FKG Dentaire, Switzerland). After that, the working length was estimated with an apex locater (SybronEndo, California, USA) and a periapical radiograph estimated with a file inserted into the canals for length confirmation.

Cleaning and shaping were performed by using IRace files (FKG Dentaire, Switzerland). The canals were irrigated during mechanical instrumentation with 10 mL of 5.25% sodium hypochlorite, followed by saline washing and then rinsing with 3 mL 17% EDTA using a 27-G side-venting needle. Passive ultrasonic irrigation (EndoActivator, Dentsply, Sirona, USA) was used to remove any debris and to permit irrigation material inside the dentinal tubules.

The canals were then partially dried using a paper point, with care taken to not dry the canals completely. Gutta-percha master cones that were 1 size smaller than the master apical file were used. BC sealer premixed TotalFill (FKG Dentaire, Switzerland) was injected under magnification with an endodontic microscope (Leica, Germany) to fill the canal, and a single master gutta-percha cone (FKG Dentaire, Switzerland) was gently (without pressure) placed into the working length.

Excess gutta-percha was removed with thermal cutting, and the remaining gutta-percha was vertically packed with a plugger. In cases with direct restoration, 3 mm premixed injectable GIC (RIVA, SDI, Austria) and universal composite (GC, Alsip, IL, USA) was used to fill the cavity, whereas in cases of indirect restoration (Endocrown), the cavity was prepared, and scanning intraoral capturing was carried out, followed by cementation with a resin-based cement (ITENA, France).

### 3.1. Clinical and Radiographic Evaluation

A recall follow-up for the patient was performed at 6 and 12 months to evaluate the obturation quality such as absence of void, presence of continuous opacity, and canal filled to the apical end and was conducted by 3 observers in separated rooms (radiologist, endodontist, and maxillofacial surgeon). Also, the evaluation included assessment of the healing of the periapical lesion as follows:

1. Complete healing: well-defined continuous lamina dura, functional and asymptomatic tooth.
2. Incomplete healing: some or no reduction of periradicular (apical) pathosis, radiolucency around the root, nonfunctional and symptomatic or asymptomatic tooth.

The clinical examination was performed by inspection, palpation, and percussion for the tooth and adjacent mucosa and included signs and/or symptoms, such as tenderness, mobility, sinus tract, periapical pocket, swelling, and history of pain were also recorded.

### 3.2. Outcome Assessment

Patient-related factors were registered: age and gender. Tooth-related factors included tooth type, position, pulpal disease, lesion size, pocket depth, sinus tract, preoperative percussion, and palpation sensitivity. Treatment-related factors included treatment type (initial treatment or retreatment), extruded sealer, and time of follow-up.

Periapical radiographs with radiolucency were divided into three subgroups:

1. Large lesion with diameter of more than 5 mm
2. Medium lesion with diameter size between 2 and 5 mm
3. Small lesion with diameter less than 2 mm

Complete healing was considered a success, whereas incomplete healing was categorized as a failure.

### 3.3. Data Analysis

For statistical analysis, a Pearson's chi-square test was used to determine the effect of each prognostic factor after the data were grouped. *p* value of ≤0.05 was considered significant. Statistical tests were performed with the SPSS v23.0 software (IBM Corp, Armonk, NY, USA).

## 4. Results

One hundred and ninety-five patients were analyzed with a mean follow-up time of 20.44 months (36 months) and an average age of 36.11 years.

The sample comprised of 143 (73.4%) female and 52 (26.6%) male patients. There were 159 (81.5%) initial patients and 36 (18.5%) retreatment cases. Patients' characteristics are summarized in Table 1.

**Figure 1:**
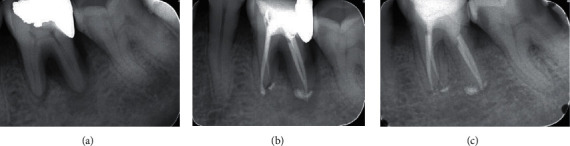
Large size lesion in initial treatment endoperio lesion. A: diagnostic X-ray; B: final obturation; C: 12 month follow-up.

**Figure 2:**
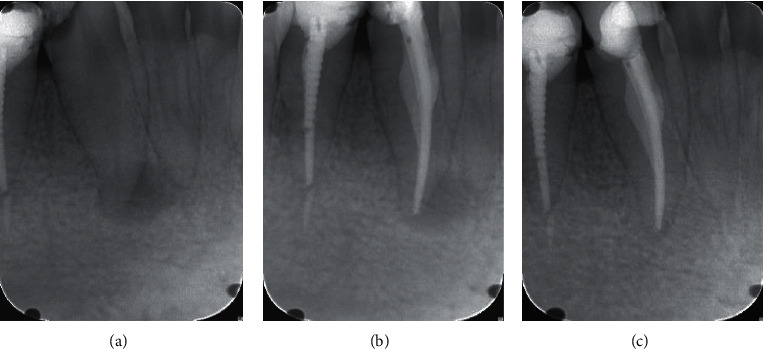
Medium-size lesion in initial treatment without extruded sealer periapical lesion. A:diagnostic X-ray; B: final obturation; C: 24 month follow-up.

**Figure 3:**
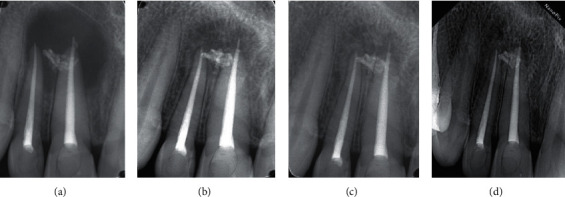
Large-size lesion in retreatment case. A: final obturation; B: 24 month follow-up; C: 36 month follow-up; D: 42 month follow-up.

In Table 2, treatment results in related factors and healing outcome were presented. The overall success rate was 84.1% (164) healed, and the rate of incomplete healing was 15.9% (31 cases). The cases with a success rate were 88.7% for initial treatment (Figures 1 and Figure 2) and 63.9% for retreatment (Figure 3).

Highly significant differences were found between the two treatment types and state of healing according to the lesion size, with small- (86.5%) and medium-size (94.6%) lesions more likely to heal in comparison with larger-size ones (73.6%).

In terms of age, a nonsignificant difference was found between younger (less than 40 years) and older patients (88% vs. 78.2%). Approximately, 134 treated teeth exhibited extrusion of the sealer, whereas 61 were not extruded. For healing in relation to the extruded sealer, no significant difference was observed.

## 5. Discussion

The goals of root canal treatment are symptom-free teeth that have normal function. Researchers have tested a variety of materials and techniques for obturation to ensure a tight seal apically and coronally which prevent bacterial ingress pathways and tooth reinfection [22–24].

In this clinical study, healing of the apical area was examined in 195 patients, with an average follow-up time of 20.44 months and a success rate of 84.1% of completely healing for 164 patients from a total of 195 total cases. In contrast, 31 (15.9%) patients were not healed.

The high percentage of healing (more than 12.5%) was most likely due to the properties of the obturation material, strong irrigation system. Bioceramic sealer is an alkaline material and has strong antimicrobial efficacy, particularly against *Enterococcus faecalis* [25] which is considered to be the most common anaerobic bacterium in the root canals [26, 27].

Increased pH may hinder the establishment of the protein motive force for the synthesis of adenosine triphosphate (ATP) [28]; also, the sealer is bioactive material that can release a significant amount of calcium minerals which lead to cell damage and inactivation of bacterial enzymes. Moreover, healing of the apical area is related to the sealer's insolubility and its bonding to the dentin and gutta-percha cone that produce sealing ability and chemical stability (hydroxyapatite formation) [29].

The second reason for the high rate of healing was most likely associated with the use of irrigation systems that include NaOCl (5.25%) and EDTA (17%), with the vibration system leading to any necrotic pulp tissue and remnant debris being dissolved efficiently. NaOCl is considered to be the most optimal irrigants for use throughout instrumentation because it possesses potent antimicrobial and proteolysis activity [30].

NaOCl ionizes into hypochlorite ions (OCl−), sodium ions (Na+), and hypochlorite acid, which has a strong effect against anaerobic microbes, particularly *E. faecalis* bacteria, and it also has a direct effect by dissolving necrotic tissues [31, 32]. EDTA (17%) was used to remove the smear layer from dentinal tubules; this layer is composed of remnants of debris collected in dentinal tubules after cleaning and shaving of necrotic dentin. It has become a media for culturing different microbial species [33].

The hydraulic condensation technique was used, which includes a single-cone gutta-percha with a silicate-based filling bioceramic sealer without any pressure. The advantages of this approach are that it is conservative, easy manipulation, time consuming, decreases the possibility of root fracture and reduces the hazard associated with heat production on the periodontal tissue, which is commonly associated with the thermoplastized warm technique [34, 35]. The biocompatible alkaline material was used as a base filling material, leading to a shift in treatment modality for large lesions and without the requirement for multiple visits and intra-canal medication.

The clinical cases which are not healed are 15.9% of patients, most cases involved retreatment which include crack propagation [36] and combined apical and lateral periodontitis [21], and these cases are mostly with persistent anaerobic bacteria and their toxins in relation to the lesion size. Also, it is observed that 18 cases are not healed in the initial treatment, most being related to patient age. Aging patients may have a substantial number of medically compromised diseases, leading to decreased immune system function.

Different pathological conditions may be responsible for periapical alterations, including 15 types that were identified following Wood's 1984 modifications [21]. Moreover, bioceramic sealers have the disadvantage that the formation of voids frequently cannot be observed in digital two-dimensional radiographs, and such areas may act as sites for bacterial proliferation [37].

In sum, a premixed injectable pure calcium silicate-based sealer with single cone gutta-percha under magnification with an endodontic microscope can improve the success rate for different lesion sizes, even those more than >5 mm in diameter.

## 6. Conclusions

Good knowledge of dental material properties and correct treatment plans with simple endodontic management are necessary for achieving a successful outcome in root canal therapy. This approach represents a shift from the classical management strategy that includes multiple, time-consuming visits by using intracanal medication seven for large-size cystic lesions.

## Figures and Tables

**Table 1:** Patients' demographic data.

Factor no./percentage							
Gender		Treatment type		Lesion size		Average age	Average recall time
Male	52 (26.6%)	Initial	159 (81.5%)	Large	72 (36.9%)	36.11 years	20.44 months
Female	143 (73.4%)	Retreatment	36 (18.5%)	Medium	56 (28.7%)		
Total	195 (100%)	Total	195 (100%)	Small	67 (34.4%)		

**Table 2:** Treatment results in related factors and healing outcome.

	Factors	Healed	Not healed	Total	*p* value
Gender	Female	123 (86%)	20 (14%)	143 (73.3%)	0.266
	Male	41 (78.8%)	11 (21.2%)	52 (26.7%)	
	Total	164 (84.1%)	31 (15.9%)	195 (100%)	

Age (years	<40	103 (88%)	14 (12%)	117 (60%)	0.066
	>40	61 (78.2%)	17 (21.8%)	78 (40%)	

Treatment type	Initial	141 (88.7%)	18 (11.3%)	159 (81.5%)	<0.001
	Retreatment	23 (63.9%)	13 (36.1%)	36 (18.5%)	

Size of lesion	Large	53 (73.6%)	19 (26.4%)	72 (36.9%)	0.004
	Medium	53 (94.6%)	3 (3.4%)	56 (28.7%)	
	Small	58 (86.5%)	9 (13.5%)	67 (34.4%)	

Extrusion of the sealer	Present	114 (85%)	20 (15%)	134 (68.7%)	0.582
	Absent	50 (81.9%)	11 (18.1%)	61 (31.3%)	

## Data Availability

The data used to support the findings of this study are available from the corresponding author upon request.
